# Road to Paris-2024: The Path of Brazilian Volleyball Athletes Goes Through the Youth National Teams

**DOI:** 10.5114/jhk/191099

**Published:** 2024-12-19

**Authors:** Elisa Dell’Antonio, Jean Marlon Machado, Felipe G. Mendes, Gabriel Henrique Treter Gonçalves

**Affiliations:** 1 Department of Physical Education, University of the State of Santa Catarina, Florianópolis, Brazil.; 2 Department of Social Sciences, University of Luxembourg, Esch-sur-Azette, Luxembourg.

**Keywords:** talent identification, athlete selection, sports success, team sport, relative age effect

## Abstract

This study analyzed the transition of Brazilian volleyball athletes from youth national teams to adult national teams, and examined the relative age effect in the selection of athletes for youth categories and their transition to the adult category. In youth categories, 326 athletes participating in the U18 and U20 women's and U19 and U21 men's world championships between 2001 and 2021 were prospectively analyzed. In the adult category, a retrospective analysis was conducted on 55 athletes who participated in the Olympic Games or World Championships between 2004 and 2022. Our results showed that approximately 20% of athletes participating in youth categories advanced to the adult national team. However, around 80% of adult teams comprised athletes who were part of youth categories. An athlete who progressed through both youth categories was 35 times more likely to be selected to the adult national teams compared to an athlete who only participated in the U18/U19 youth team. In youth national teams, there was a higher call-up rate for athletes born earlier. Despite this, athletes born later were more likely to advance to the adult national team.

## Introduction

The process of training and selecting athletes is a constant subject of investigation across various sports disciplines (Baker et al., 2020; Pino-Ortega et al., 2021). When examining successful athletes, the goal is to identify characteristics, contexts, and circumstances that contribute to high athletic performance, especially in adulthood (Güllich et al., 2022).

Recent studies (Barth et al., 2022; Güllich et al., 2022, 2023a) suggest that adult athletes who compete at an international level were children or teenagers who engaged in various sports, started later in the sport they currently compete in, and progressed more slowly than their national-level counterparts. Furthermore, when focusing specifically on the volleyball scenario, evidence from various countries underscores the importance of anthropometric characteristics and physical performance in the athlete selection process (Gabbett and Georgieff, 2007; Mendes et al., 2021; Milić et al., 2017; Sheppard et al., 2012). Among these variables, those that stand out are the attack range, height, and jump height (Mostaert et al., 2022; Schons et al., 2022; Tsoukos et al., 2019).

It is currently accepted that, across various sports disciplines, successful youth and adult athletes generally represent distinct populations. Güllich et al. (2023b) prospectively reported that approximately 90% of U17/18 international-level athletes did not reach the same level in the adult category. Successful young athletes often start practicing their main sport at an early age and tend to progress more quickly at the beginning of their career. However, excessively specialized practice does not seem to lead these athletes to a high level of sports performance in adulthood (Barth et al., 2022; Güllich et al., 2023a). It is also worth considering that, when analyzing the major competitions in the world of sports, such as world championships, each athlete typically participates in one, or at most two, editions of each event in the youth categories. Meanwhile, in the adult category, a top-tier athlete can remain competitive for more than a decade, limiting the opportunities for new athletes to enter the scene. As an example, we can cite the Brazilian volleyball teams, which, in the last five editions of the Olympic Games and World Championships (between 2004 and 2022), utilized 49 female and 49 male athletes out of possible 124 in each category.

Retrospectively, it has been shown that over 80% of elite adult athletes were not successful youth athletes (Güllich et al., 2023b), highlighting the complex and non-linear nature of the processes involved in sports training and selection (Abbott and Collins, 2004; Till and Baker, 2020). Therefore, it is necessary to investigate specific disciplines and countries, in order to understand the path that athletes have taken in their training and identify contributions that can be made to improve these processes. Additionally, the comprehensive scoping review conducted by Baker et al. (2020) highlighted that these studies were predominantly focused on male soccer players, suggesting that investigations in other contexts would be beneficial.

Considering volleyball, adult national teams have a packed schedule, with special emphasis on the quadrennial events, the Olympic Games and the World Championships. Youth national teams, which include U19 and U21 categories for men and, as of 2023, for women as well (previously U18 and U20), participate biennially; in one year they compete in the continental championship, which serves as a qualifier for the world championship the following year. The primary reason for grouping ages in youth categories is to minimize the differences in cognitive and physical development and ensure a more equitable competition (Campos et al., 2020). However, within the same age group, there is evidence of a priority in selecting chronologically older athletes. This phenomenon is known as the Relative Age Effect (RAE) and has been studied across various sports disciplines (Bezuglov et al., 2024; Brustio et al., 2023a; Gómez-López et al., 2017; Lemoyne et al., 2023; López de Subijana and Lorenzo, 2018; Ribeiro Junior et al., 2021), including volleyball (Campos et al., 2020; Campos et al., 2016; Rubajczyk and Rokita, 2020; Sliwa et al., 2021).

Several studies have shown that RAE is present in the careers of youth volleyball players (Campos et al., 2020; Okazaki et al., 2011; Rubajczyk and Rokita, 2020; Sliwa et al., 2021), nevertheless, there is evidence indicating that RAE is lower during their adult career (Brustio et al., 2022; Campos et al., 2016; Lupo et al., 2019; Parma and Penna, 2018). Another relevant aspect is that despite recognizing the importance of anthropometric characteristics and physical performance in the selection process for athletes in the sport (Mostaert et al., 2022; Schons et al., 2022; Tsoukos et al., 2019), there is not a big variation among these characteristics within competitive age groups. As such, there seems to be no relationship between a birth trimester and height, jump height and the attack range (Papadopoulou et al., 2019; Sliwa et al., 2021).

The international recognition of Brazilian volleyball, which has shown significant and consistent results across various competitions over the last three decades (with six titles, eight silver medals, and two bronze medals out of 20 contested medals in the Olympic Games and World Championships from 2004 to 2022), positions Brazil as a benchmark in the development of athletes in the sport and presents an important context for research. In view of the above, the aim of the current study was to analyze the transition of Brazilian athletes from youth teams to adult national teams. Additionally, the specific objectives of this study were to examine RAE in the selection processes of youth category athletes and to assess the influence of RAE on the transition process to the adult national team.

## Methods

### Participants

Athletes who participated in the U18 women's and U19 men's World Championships from 2001 to 2019, and/or the U20 women's and U21 men's championships from 2003 to 2021 were considered. This amounted to a total of 480 call-ups, involving 327 athletes (159 women, with individual data for one athlete unavailable, and 168 men). Out of the 326 athletes analyzed, 97 were selected only for the U18 and U19 teams, 91 were selected only for the U20 and U21 teams, and 138 were selected for both categories (U18/U20 and U19/U21). The criterion for success in the adult category was participation in an edition of the Olympic Games or the Senior World Championship from 2004 to 2022. From 248 call-ups, a total of 98 athletes (49 female and 49 male) were included, with 28 women and 27 men being eligible, based on their birth dates (women born in 1984 or later and men born in 1983 or later), for the youth categories during the period selected for this study.

### Design and Procedures

The data were extracted from the websites of the International Volleyball Federation (FIVB) (www.fivb.com) and the Brazilian Volleyball Confederation (CBV) (www.cbv.com.br). The athlete's date of birth was included, and semi-annual benchmarks were established starting from the year of the athlete's first participation in a youth world championship (born between January and June of the last eligible year = S1; between July and December of the last eligible year = S2; between January and June of the penultimate eligible year = S3; and from July of the penultimate eligible year = S4). Biannual benchmarks were adopted because the youth world championships occur every two years.

### Statistical Analysis

We conducted a retrospective descriptive analysis as well as a prospective analysis. For the retrospective analysis, starting from the list of athletes who achieved success in the adult category, we identified those who, earlier in their careers, had participated in the world championships in the youth categories (U18/19 or U20/21). For the prospective analysis, starting from the list of athletes who had participated in the world championships in the youth categories, we identified those who achieved success in the adult category later in their careers. All the study data were in the public domain, therefore, informed consent was not required.

To examine the association between success in the adult category and participation in the youth categories, as well as the relationship between success in the adult category and relative age in the year of the first call-up to the youth category, we used the χ^2^ independence test. Due to the lack of data regarding athletes who did not achieve success in the adult category and did not participate in any youth categories, the data from athletes who were successful in the adult category, but did not participate in any youth categories were excluded from these association analyses (n = 4 females and 3 males). The Effect Size (ES) of the χ^2^ independence test was determined based on the value of Cramer's V. Cramer's V of 6% or less was considered a trivial ES; between 6% and 17%, small; between 17% and 29%, medium; and 29% or greater, a large ES. We also calculated the Odds Ratio (OR) and the respective 95% confidence intervals for each association identified. In instances where the count was equal to zero, the OR was calculated using the Haldane-Anscombe correction. The tests were conducted using SPSS software, version 20.0, and Microsoft Office Excel, version 2312, with a *p*-value set at ≤ 0.05. All analyses were conducted for the total sample and stratified by sex.

## Results

**Figure 1:**
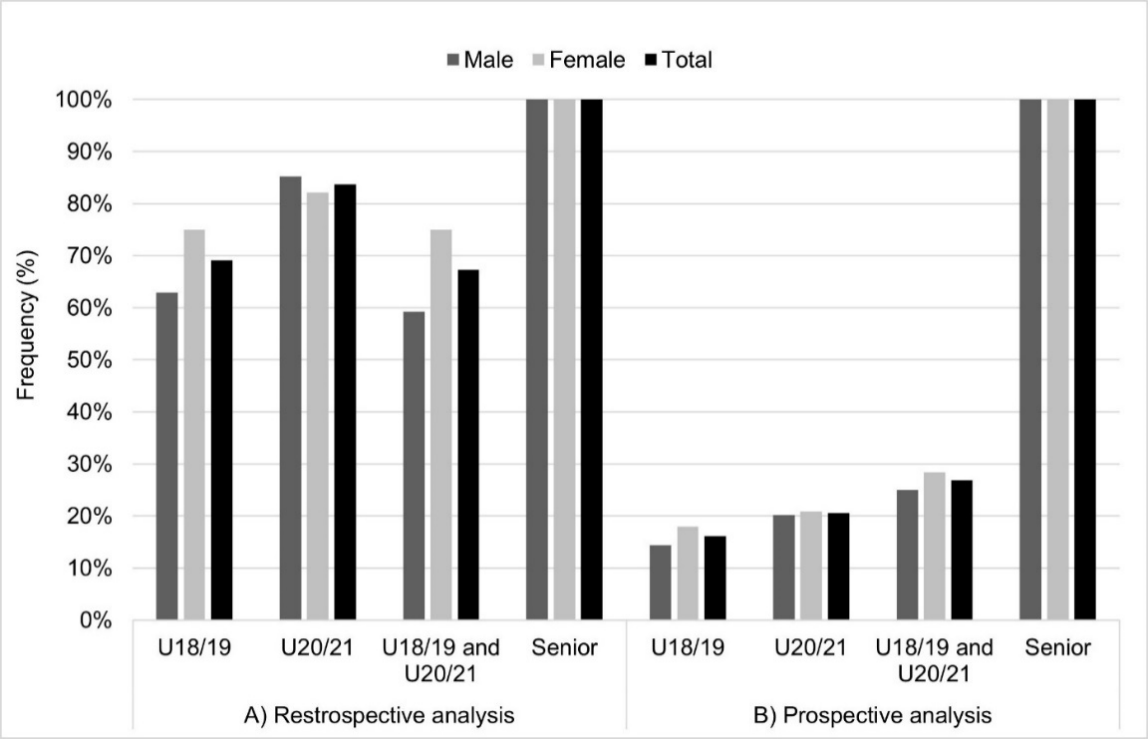
Analysis of the transition of athletes between youth categories (U18/19 and U20/21) and the senior category, based on retrospective and prospective analysis.

The most successful athletes in the adult category also participated in the youth categories. Among athletes who participated in the Olympic Games or Senior World Championships from 2004 to 2022, over 80% (46/55) had competed in the U20/21 category, nearly 70% (38/55) in the U18/19 category, and more than 60% (37/55) in both of the aforementioned categories earlier in their careers (Figure 1A). Regarding the prospective analysis, 16% (38/235) of athletes who participated in the U18/19 category, 21% (47/229) of athletes who participated in the U20/21 category, and 27% (37/138) of athletes who participated in both categories were later called up to participate in an edition of the Olympic Games and/or the Senior World Championships (Figure 1B).

The associations between success in the adult category, participation in the youth categories, and relative age in the year of the first call-up to a youth category can be observed in Table 1. The results of the χ^2^ test of independence showed associations of 31.1%, 27.6%, and 35.3% between being called up for the adult category and participation in the youth categories for the total sample (χ^2^[2] = 31.555; *p* < 0.001) and when stratified by sex, for males (χ^2^[2] = 12.821; *p* = 0.002) and females (χ^2^[2] = 19.665; *p* < 0.001), respectively. For the entire sample, athletes who participated only in the U20/21 category were 11.85 times more likely to be called up for participation in the adult category than athletes who only competed in the U18/19 category. Athletes who participated in both youth categories (U18/19 and U20/21) were 35.17 times more likely to be called up to the adult national team compared to athletes who only participated in the U18/19 category.

Furthermore, there was a significant association of 15.6% between being called up for the adult category and relative age in the year of the first call-up for the youth category, across the entire sample (χ^2^[3] = 7.952; *p* = 0.047). Athletes who were called up for their first participation in the youth category and who were born in the second half of the penultimate eligible year, had a threefold greater chance of being subsequently selected for the adult category compared to athletes born in the first half of the last eligible year.

**Table 1:** Associations between participation in the youth and adult categories.

	Adult category		Total n (%)	*p*-value*	Cramer’s V (%)	OR (CI95%)
	Called up n (%)	Not called up n (%)				
**Total sample**						
Youth categories						
U18/19	1 (1)	96 (99)	97 (100)	< 0.001	31.1	1 (ref)
U20/21	10 (11)	81 (89)	91 (100)			11.85 (1.49–94.57)
U18/19 and 20/21	37 (26.8)	101 (73.2)	138 (100)			35.17 (4.73–261.39)
Birth semester						
S1	16 (10.7)	134 (89.3)	150 (100)	0.047	15.6	1 (ref)
S2	9 (12.2)	65 (87.8)	74 (100)			1.16 (0.49–2.76)
S3	14 (20.6)	54 (79.4)	68 (100)			2.17 (0.99–4.75)
S4	9 (26.5)	25 (73.5)	34 (100)			3.02 (1.2–7.58)
**Male**						
Youth categories						
U19	1 (1.9)	53 (98.1)	54 (100)	0.002	27.6	1 (ref)
U21	7 (14)	43 (86)	50 (100)			8.63 (1.02–72.86)
U19 and 21	16 (25)	48 (75)	64 (100)			17.67 (2.26–138.3)
Birth semester						
S1	8 (9.2)	79 (90.8)	87 (100)			1 (ref)
S2	3 (9.4)	29 (90.6)	32 (100)			1.02 (0.25–4.12)
S3	9 (26.5)	25 (73.5)	34 (100)			3.56 (1.24–10.19)
S4	4 (26.7)	11 (73.3)	15 (100)			3.59 (0.93–13.93)
**Female**						
Youth categories						
U18	0 (0)	43 (100)	43 (100)	< 0.001	35.3	1 (ref)⊥
U20	3 (7.3)	38 (92.7)	41 (100)			7.91 (0.4–158.04)⊥
U18 and 20	21 (28.4)	53 (71.6)	74 (100)			34.96 (2.06–593.83) ⊥
Birth semester						
S1	8 (12.7)	55 (87.3)	63 (100)			1 (ref)
S2	6 (14.3)	36 (85.7)	42 (100)			1.15 (0.37–3.58)
S3	5 (14.7)	29 (85.3)	34 (100)			1.19 (0.36–3.95)
S4	5 (26.3)	14 (73.7)	19 (100)			2.46 (0.7–8.67)

n: absolute frequency; %: relative frequency; *: χ^2^ independence test; OR: Odds ratio; CI95%: 95% confidence interval; ref: reference category; ⊥: Odds ratio calculated using the Haldane-Anscombe correction;: χ^2^ independence test could not be run due to expected counts lower than 5; S1/S2/S3/S4: Birth semester from the athlete's first participation in youth categories

## Discussion

The key findings of this study suggest that in Brazilian volleyball, ~20% of athletes who competed in world championships in the youth categories participated in adult national teams in the major competitions on the calendar. Although it may be considered low, this figure is similar to that found by Brustio et al. (2023a) and Boccia et al. (2023) in the context of women’s and men's football teams in Italy, respectively. Furthermore, it is above the average reported for the professionalization of Brazilian basketball players in the country's first league (Ribeiro Junior et al., 2021), as well as for other sports disciplines worldwide (~10%) (Güllich et al., 2023b). It is worth noting that the presented transition rate from the youth to adult categories could be higher if we had adopted less restrictive criteria and also included participation in other youth calendar competitions (such as continental championships), as well as if we expanded inclusion in the adult national team to all official events (annual competitions, such as the Volleyball Nations League and continental championships). Barreiros et al. (2014) highlight that approximately 55% of male players (15 out of 27) and 22% of female players (6 out of 27) from Portugal's U16 volleyball teams were later selected for the adult national team. It is important to note that the selection process for the youth categories considered 16 events for men and 2 events for women, while for the adult category, 140 events for men and 60 events for women were taken into account.

Despite the participation criteria established for the eligibility of the athletes analyzed in this study (in total, for both men and women, 20 youth category events and 10 adult category events were considered), one factor that may enhance the transition rate presented is the existence of youth categories with older age groups (U18/19 and U20/21). If we had included youth categories with younger ages, the transition rate would likely be reduced, since the earlier the start, the more challenging it becomes to accurately identify talent. For example, a study conducted in Belgium followed 1,758 volleyball athletes aged between 10 and 13 years and found that only 52 (3.0%) players advanced to the youth national teams (Mostaert et al., 2022). Furthermore, according to the literature and our data, approximately 10 will advance to the adult national teams (~0.6% of the 1,758).

Another relevant aspect is the distribution of athletes who achieve success in the transition from the youth to adult categories across different generations of the youth categories, for which we found similar averages between women and men (2.4 ± 1.0 and 2.4 ± 1.3 athletes per generation for women and men, respectively, representing 16.4% [95% CI 11.9–20.8] for women and 14.5% [95% CI 8.2–20.7] for men). Although the results of the youth teams are significant (with five titles and additional eight semifinals for women, and four titles plus six semifinals for men), this does not seem to influence the transition rate per generation. It is also worth noting that, considering the average time between the last call-up to the youth national team and the first participation in adult competitions (5.1 ± 3.5 and 5.8 ± 3.2 years for women and men, respectively) and the average age at first participation in the adult national team (23.8 ± 3.9 and 25.2 ± 3.3 years for women and men, respectively), it is likely that several more athletes make the transition to the adult category in events such as the Paris 2024 Olympic Games.

Interestingly, our data show that adult Brazilian volleyball teams, in World Championships and the Olympic Games, comprise predominantly (> 80%) athletes who were part of the youth national teams in world championships. Taking into account only the U18/19 category, we found that ~70% of adult athletes competed in the world championships of that age group, significantly contradicting the findings of previous studies with soccer players (~40%) and athletes from various sports disciplines (~18%) (Boccia et al., 2023; Güllich et al., 2023b). Even though we are comparing different contexts of sports practices, involving distinct modalities in countries with varying numbers of players, as well as different forms of organization and sports cultures, this seems to indicate that Brazilian volleyball primarily follows a linear development pattern when considering participation in youth national teams.

Reinforcing the apparent linearity in the development process, our findings suggest that athletes who participate in world championships exclusively in the U18/19 categories are less likely to be called up to the adult national team compared to those who are only selected for the U20/21 categories or those who accumulate selections in both youth categories. This means that if an athlete is called up to the first youth category and then fails to be selected for the next category, they have essentially been dropped from the development process (they have been forgotten) and have little chance of getting back on track and advance to the adult national team. A single call-up in the U20/21 category appears to be more promising, as, albeit late, it places the athlete on track and increases their chance of being selected for the adult national team by nearly twelvefold compared to athletes who only receive one call-up in the younger category. More than this, athletes who present a fairly linear path, participating in world championships across both categories, demonstrate increased chances of being called up to the adult national team, of approximately 35 times. This demonstrates the linearity of Brazilian volleyball in a global context where, across various sports disciplines, such linearity is not the norm (Abbott and Collins, 2004; Barreiros et al., 2013).

One hypothesis to explain this scenario is to assume that there is a high-quality selection process in the youth categories, and indeed, the athletes chosen are those with great potential and promising future. However, taking into account the pillars proposed by the Sports Policy factors Leading to International Sporting Success (SPLISS) model (De Bosscher et al., 2006), one might also consider that, as a result of joining youth teams, these athletes gain access to better training facilities, work with top-tier coaches, compete in a higher number of games at a more advanced level, including international competitions, have greater financial support, and thus, they are in a better position to reach their full potential in sports. Meanwhile, young athletes who are not called up for the youth national teams do not have the same opportunities and face a longer journey ahead. Therefore, it is crucial to consider the number of athletes with potential who might be overlooked because they are unable, for whatever reason, to gain access to the Brazilian youth national teams. Additionally, it seems particularly relevant to explore the journeys of the athletes (a total of seven, comprising four women and three men) who made it to the adult national team without competing in the world championships in any youth category.

Regarding the RAE, previous studies have shown that in volleyball, athletes born in the first quarters of the year have an advantage in the selection process for youth categories (Campos et al., 2020; Okazaki et al., 2011; Rubajczyk and Rokita, 2020; Sliwa et al., 2021) and for professional adult players, mostly in the beginning of senior career (Brustio et al., 2022; Lupo et al., 2019). This finding aligns with our research (46% of athletes were born in the first half of the last eligible year, while only 10.4% were born in the second half of the penultimate year). However, when we examined the successful transition from youth categories to the adult category, taking into account the entire sample, we could identify that athletes born in the second half of the penultimate eligible year who were called up for the youth categories had approximately three times the chance of being selected for the adult national team compared to those born in the first half of the last eligible year. Corroborating our results, investigations into the transition to the adult category in several sports (Lupo et al., 2019) and women's football team in Italy have also reported a higher transition rate for relatively younger athletes (Brustio et al., 2023a). A potential explanation for this can be found in the underdog hypothesis (Brustio et al., 2023b; McCarthy et al., 2016). This theory suggests that in some competitive situations, individuals or teams considered 'underdogs', meaning less capable or less likely to win, may actually have a psychological advantage over their stronger opponents. Although the mechanisms behind this are not fully understood, this hypothesis suggests, across various sports, higher likelihood of successful transition for younger athletes. Even though the influence of biological maturation in the selection process of young volleyball athletes is recognized (Albaladejo-Saura et al., 2023), in the investigated youth categories (U18, U19, U20, and U21) it is expected that all athletes are biologically mature, and an athlete who has enough qualities to be selected for the world championship in these youth categories, being approximately 1.5 years younger than the competition's age limit, appears to be a sporting talent with a good chance of maintaining this level and continuing their career by being selected for the adult national team.

Rubajczyk et al. (2020) identified RAE in over 4,900 young Polish athletes (~14/15 years old) participating in the National Volleyball Development Program (~70% of the participants were born in the first half of the year, and 6% of the males and 11% of the females were born in the last quarter of the year). No differences were found in the anthropometric and physical characteristics among the selected players, regardless of their birth quartile. Additionally, the authors identified differences between selected and non-selected athletes, emphasizing the significance of height and attack reach in the selection process of young volleyball players.

Also in Poland, the study by Sliwa et al. (2021), which involved 232 elite young male volleyball players from the Sports School, examined the RAE and reported that the lowest proportion of athletes was born in the last quarter of the year. This was true for both the overall sample (8.2%) and the separate samples considering athletes who completed their training (n = 177) (9.0%) and those who did not (n = 55) (5.4%). Athletes born in the last quarter of the year who completed their training did not differ from their peers in terms of height, body mass, and attack reach.

Therefore, it is crucial to grasp the significance of anthropometric and physical factors in the selection process and in distinguishing between the competitive levels of volleyball players (Jahandideh et al., 2021; Pocek et al., 2020; Rubajczyk and Rokita, 2020; Tsoukos et al., 2019), even though these are not the sole determining aspects (Mostaert et al., 2022). Tsoukos et al. (2019) highlighted differences in physical capabilities between selected and non-selected young athletes (~15 years old), noting that selected athletes demonstrated superior performance. Furthermore, the authors emphasized that the attack reach could explain more than 50% of the selection process. Jahandideh et al. (2021) conducted a study on 240 athletes participating in an U18 World Championship edition and reported that the top four teams were taller, heavier, and had a greater attack and block reach compared to teams ranked 5^th^ to 20^th^. Additionally, Mostaert et al. (2022), in their research on the selection process with youth volleyball athletes (~11/12 years old), beyond height and vertical jump performance, emphasized the importance of motor coordination, especially for female athletes. Futhermore, for Olympic athletes across various sports disciplines, Issurin (2017) emphasized that the appropriate physique, high trainability/learning potential, and physiological requirements, along with personality traits and the early acquisition of psychological skills, were crucial for athletic success.

We consider the criteria used for selecting the sample from the youth categories and the adult category as a limitation of this study. We believe that broader criteria could yield different results and suggest alternative approaches to the selection and transition process of athletes in Brazilian volleyball. Additionally, we emphasize that transitioning to the adult national team should not be the only measure of success, but rather the benchmark chosen for this study. We understand that many athletes can also have successful professional careers in sports and beyond without competing in an edition of the Olympic Games or a Senior World Championship.

## Conclusions

The present study offers significant contributions, as less than 4% of all research on sports talents investigate volleyball, and fewer than 2% of the total involve samples of Brazilian athletes (Baker et al., 2020).

Our results indicate that approximately 20% of Brazilian volleyball athletes who compete in youth world championships advance to adult national teams in World Championships and/or the Olympic Games. Additionally, adult teams in these competitions are composed of approximately 80% of athletes who were part of youth categories. We also found that an athlete who competed in the world championships in both categories was approximately 35 times more likely to be called up to the adult national team than an athlete who had only one call-up in the U18/19 category, which indicates a linear pattern of development.

Regarding the RAE, we found that athletes born in the first half of the last eligible year for competition were more frequently selected for youth national teams. However, when we examined the transition to the adult national team, our data showed that athletes born in the second half of the penultimate year were approximately three times more likely to succeed in this transition compared to athletes born in the first half of the last year.

It is suggested that future studies investigate how the transition process from youth categories to adult national teams occurs in other countries that are powerhouses in the sport, as well as examine athletes‘ performance in competitions and/or variation between players’ positions. In addition, it would be interesting to consider the access processes to youth national teams in Brazil and gain deeper understanding of the perspectives and involvement of coaches in this process.

## References

[ref1] Abbott, A. and Collins, D. (2004). Eliminating the dichotomy between theory and practice in talent identification and development: considering the role of psychology. Journal of Sports Sciences, 22(5), 395–408. 10.1080/02640410410001675324 15160593

[ref2] Albaladejo-Saura, M., Vaquero-Cristóbal, R., García-Roca, J. A., Esparza-Ros, F. (2023). What Variables Allow the Differentiation between More and Less Successful Adolescent Volleyball Players?. Journal of Human Kinetics, 88, 229–242. 10.5114/jhk/166107 PMC10407323 37559765

[ref3] Baker, J., Wilson, S., Johnston, K., Dehghansai, N., Koenigsberg, A., de Vegt, S. and Wattie, N. (2020). Talent research in sport 1990–2018: a scoping review. Frontiers in Psychology, 11, 607710. 10.3389/fpsyg.2020.607710 PMC7723867 33324305

[ref4] Barreiros, A., Côté, J. and Fonseca, A. M. (2013). About development of talent in sport: a contribution from theoretical models of sport development. Revista de Psícologia Del Deporte, 22(2), 489–494.

[ref5] Barreiros, A., Côté, J. and Fonseca, A. M. (2014). From early to adult sport success: Analysing athletes’ progression in national squads. European Journal of Sport Science, 14(Suppl 1), S178–82. 10.1080/17461391.2012.671368 24444203

[ref6] Barth, M., Güllich, A., Macnamara, B. N. and Hambrick, D. Z. (2022). Predictors of junior versus senior elite performance are opposite: a systematic review and meta-analysis of participation patterns. Sports Medicine, 52(6), 1399–1416. 10.1007/s40279-021-01625-4 35038142 PMC9124658

[ref7] Bezuglov, E., Semeniuk, N., Shoshorina, M., Savin, E., Waśkiewicz, Z., Emanov, A., Malyakin, G., Telyshev, D. and Morgans, R. (2024). Is There a Relative Age Effect among the Most Successful Track and Field Athletes?. Journal of Human Kinetics, 92, 193–202. 10.5114/jhk/174497 38736604 PMC11079924

[ref8] Boccia, G., Brustio, P. R., Rinaldi, R., Romagnoli, R., Cardinale, M. and Piacentini, M. F. (2023). Junior to senior transition pathway in Italian Football: the rocky road to the top is not determined by youth national team’s selections. *PloS One*, 18(7), e0288594. 10.1371/journal.pone.0288594 PMC10353809 37463153

[ref9] Brustio, P. R., Boccia, G., De Pasquale, P., Lupo, C. and Ungureanu, A. N. (2022). Small relative age effect appears in professional female italian team sports. International Journal of Environmental Research and Public Health, 19, 385. 10.3390/ijerph19010385 PMC8750980 35010643

[ref10] Brustio, P. R., Modena, R., Boccia, G., Vogliazzo, M. and Kelly, A. L. (2023a). Youth-to-senior transition in women’s and girls’ football: towards a better understanding of relative age effects and gender-specific considerations. *PLoS ONE*, 18(5), e0283781. 10.1371/journal.pone.0283781 PMC10159103 37141307

[ref11] Brustio, P. R., Stival, M. and Boccia, G. (2023b). Relative age effect reversal on the junior-to-senior transition in world-class athletics. Journal of Sports Sciences, 41(9), 903–909. 10.1080/02640414.2023.2245647 37555554

[ref12] Campos, F. A. D. C., Stanganelli, C. L. R., Rabelo, F. N., Campos, L. C. B. and Pellegrinotti, Í. L. (2016). The relative age effect in male volleyball championships. International Journal of Sports Science, 6(3), 116–120. 10.5923/j.sports.20160603.08

[ref13] Campos, F. A. D., Pellegrinotti, Í. L., Campos, L. C. B., Dias, T. M. R. and Gómez, M.-Á. (2020). Relative age effect in the Girls’ Volleyball U18 World Championship. Journal of Human Kinetics, 72(1), 195–202. 10.2478/hukin-2019-0106 32269660 PMC7126263

[ref14] De Bosscher, V., De Knop, P., Van Bottenburg, M. and Shibli, S. (2006). A conceptual framework for analysing sports policy factors leading to international sporting success. European Sport Management Quarterly, 6(2), 185–215. 10.1080/16184740600955087

[ref15] Gabbett, T. and Georgieff, B. (2007). Physiological and anthropometric characteristics of Australian junior national, state, and novice volleyball players. Journal of Strength and Conditioning Research, 21(3), 902–908. 17685708 10.1519/R-20616.1

[ref16] Gómez-López, M., Granero-Gallegos, A., Molina, S. F. and Ríos, L. J. C. (2017). Relative age effect during the selection of young handball player. Journal of Physical Education and Sport, 17(1), 418–423. 10.7752/jpes.2017.01062

[ref17] Güllich, A., Barth, M., Hambrick, D. Z. and Macnamara, B. N. (2023a). Participation patterns in talent development in youth sports. *Frontiers in Sports and Active Living*, 5, 1175718. 10.3389/fspor.2023.1175718 PMC10232881 37274619

[ref18] Güllich, A., Barth, M., Macnamara, B. N. and Hambrick, D. Z. (2023b). Quantifying the extent to which successful juniors and successful seniors are two disparate populations: a systematic review and synthesis of findings. Sports Medicine, 53(6), 1201–1217. 10.1007/s40279-023-01840-1 37022588 PMC10185603

[ref19] Güllich, A., Macnamara, B. N. and Hambrick, D. Z. (2022). What makes a champion? Early multidisciplinary practice, not early specialization, predicts world-class performance. Perspectives on Psychological Science, 17(1), 6–29. 10.1177/1745691620974772 34260336

[ref20] Issurin, V. B. (2017). Evidence-based prerequisites and precursors of athletic talent: a review. Sports Medicine, 47(10), 1993–2010. 10.1007/s40279-017-0740-0 28493064

[ref21] Jahandideh, A., Rohani, H. and Hemmati, S. (2021). Anthropometric profile of FIVB Volleyball Girls’ U18 World Championship volleyball players according to the playing position-World Championship 2017. International Journal of Sport, Exercise and Health Research, 5(1), 23–27. 10.31254/sportmed.5107

[ref22] Lemoyne, J., Trudeau, F., Grondin, S. (2023). The Relative Age Effect in Ice Hockey: Analysis of Its Presence, Its Fading and of a Reversal Effect among Junior and Professional Leagues. Journal of Human Kinetics, 87, 119–131. 10.5114/jhk/161573 PMC10203842 37229406

[ref23] López de Subijana, C. and Lorenzo, J. (2018). Relative age effect and long-term success in the spanish soccer and basketball national teams. Journal of Human Kinetics, 65(1), 197–204. 10.2478/hukin-2018-0027 30687431 PMC6341957

[ref24] Lupo, C., Boccia, G., Ungureanu, A. N., Frati, R., Marocco, R. and Brustio, P. R. (2019). The beginning of senior career in team sport is affected by relative age effect. *Frontiers in Psychology*, 10, 1465. 10.3389/fpsyg.2019.01465 31293489 PMC6606777

[ref25] McCarthy, N., Collins, D. and Court, D. (2016). Start hard, finish better: further evidence for the reversal of the RAE advantage. Journal of Sports Sciences, 34(15), 1461–1465. 10.1080/02640414.2015.1119297 26651240

[ref26] Mendes, F. G., Lima, A. B., Christofoletti, M., Quinaud, R. T., Collet, C., Gonçalves, C. E. and Carvalho, H. M. (2021). Multidimensional characteristics of young Brazilian volleyball players: a Bayesian multilevel analysis. *PLoS ONE*, 16(4), e0250953. 10.1371/journal.pone.0250953 PMC8087100 33930069

[ref27] Milić, M., Grgantov, Z., Chamari, K., Ardigò, L. P., Bianco, A. and Padulo, J. (2017). Anthropometric and physical characteristics allow differentiation of young female volleyball players according to playing position and level of expertise. Biology of Sport, 34(1), 19–26. 10.5114/biolsport.2017.63382 28416892 PMC5377555

[ref28] Mostaert, M., Pion, J., Lenoir, M. and Vansteenkiste, P. (2022). A retrospective analysis of the national youth teams in volleyball: were they always faster, taller, and stronger? Journal of Strength and Conditioning Research, 36(9), 2615–2621. 10.1519/JSC.0000000000003847 33044360

[ref29] Okazaki, F. H. A., Keller, B., Fontana, F. E. and Gallagher, J. D. (2011). The relative age effect among female Brazilian youth volleyball players. Research Quarterly for Exercise and Sport, 82(1), 135–139. 10.1080/02701367.2011.10599730 21462694

[ref30] Papadopoulou, S. D., Papadopoulou, S. K., Rosemann, T., Knechtle, B. and Nikolaidis, P. T. (2019). Relative age effect on youth female volleyball players: a pilot study on its prevalence and relationship with anthropometric and physiological characteristics. *Frontiers in Psychology*, 10, 2737. 10.3389/fpsyg.2019.02737 31849799 PMC6901434

[ref31] Parma, J. O. and Penna, E. M. (2018). The relative age effect on Brazilian elite volleyball. Journal of Physical Education, 29, e2942. 10.4025/jphyseduc.v29i1.2942

[ref32] Pino-Ortega, J., Rojas-Valverde, D., Gómez-Carmona, C. D. and Rico-González, M. (2021). Training design, performance analysis and talent identification—a systematic review about the most relevant variables through the principal component analysis in soccer, basketball and rugby. International Journal of Environmental Research and Public Health, 18, 2642. 10.3390/ijerph18052642 33807971 PMC7967544

[ref33] Pocek, S., Vukovic, J., Jaksic, D., Lakicevic, N., Messina, G., Bianco, A. and Drid, P. (2020). Fitness profile of young female volleyball players. Medicina Dello Sport, 73(2), 197–209. 10.23736/S0025-7826.20.03698-4

[ref34] Ribeiro Junior, D. B., Werneck, F. Z., Oliveira, H. Z., Panza, P. S., Ibáñez, S. J. and Vianna, J. M. (2021). From talent identification to Novo Basquete Brasil (NBB): multifactorial analysis of the career progression in youth brazilian elite basketball. *Frontiers in Psychology*, 12, 6175-63. 10.3389/fpsyg.2021.617563 PMC8007766 33796044

[ref35] Rubajczyk, K. and Rokita, A. (2020). The relative age effect and talent identification factors in youth volleyball in Poland. *Frontiers in Psychology*, 11, 1445. 10.3389/fpsyg.2020.01445 32733325 PMC7358257

[ref36] Schons, P., Berriel, G. P., Preissler, A. A. B., Caporal, G. C., Costa, R. R., da Silva, L. C. R. and Kruel, L. F. M. (2022). Mathematical models to identify high-performance players for the Brazilian under-19 men’s volleyball team. Journal of Sports Sciences, 40(13), 1458–1466. 10.1080/02640414.2022.2085439 35678190

[ref37] Sheppard, J. M., Nolan, E. and Newton, R. U. (2012). Changes in strength and power qualities over two years in volleyball players transitioning from junior to senior national team. Journal of Strength and Conditioning Research, 26(1), 152–157. 10.1519/JSC.0b013e31821e4d5b 22193341

[ref38] Sliwa, M., Sadowski, J. and Buszta, M. (2021). Relative age effect and talent identification in youth volleyball players from the polish volleyball federation sports school. Polish Journal of Sport and Tourism, 28(4), 21–25. 10.2478/pjst-2021-0022

[ref39] Till, K. and Baker, J. (2020). Challenges and [possible] solutions to optimizing talent identification and development in sport. *Frontiers in Psychology*, 11, 664. 10.3389/fpsyg.2020.00664 PMC7174680 32351427

[ref40] Tsoukos, A., Drikos, S., Brown, L. E., Sotiropoulos, K., Veligekas, P. and Bogdanis, G. C. (2019). Anthropometric and motor performance variables are decisive factors for the selection of junior national female volleyball players. Journal of Human Kinetics, 67(1), 163–173. 10.2478/hukin-2019-0012 31523315 PMC6714358

